# Determining the direction of pro-safety activities using selected methods of statistical analysis

**DOI:** 10.3389/fpubh.2026.1752399

**Published:** 2026-03-20

**Authors:** Tomasz Małysa, Jacek Chrapoński

**Affiliations:** Faculty of Materials Engineering, Silesian University of Technology, Katowice, Poland

**Keywords:** accidents at work, occupational health and safety, Pearson linear correlation, risk reduction measures, steel sector

## Abstract

**Objective:**

The aim of this study was to determine the strength of the correlation between the studied characteristics describing accident events.

**Methods:**

Statistical methods were used for the analysis, particularly Pearson’s linear correlation analysis. The study was conducted using PQStat v.1.6.8 statistical software. This study present an analysis of occupational activity and causes of accidents in the studied seniority groups, using Pearson’s linear correlation analysis. Determining the strength of correlation, rather than causality, allows for the identification of key characteristics of accidents (e.g., cause of accident, type of activity performed) and the connections between the studied characteristics.

**Results:**

The study identified key activities performed by injured workers and causes of accidents in the studied seniority groups, which should form the basis for actions aimed at reducing the number of recorded occupational accidents. To this end, the strongest correlations in the seniority group-activity-cause of accident pattern are presented graphically.

**Conclusion:**

Determining the strongest correlations allows for the identification of key areas on which actions aimed at improving occupational safety should be based. These actions may therefore lead to a reduction in the number of recorded accidents in particular age groups in which the strongest connections between the studied characteristics were identified.

## Introduction

Accidents at work are events whose occurrence is associated with costs that organizations incur in recording them (1–3). Definitions of occupational accidents vary, depending on the legislation of the country in which the events are recorded. Consistency in terms of definitions is found in the fact that an accident is a sudden event caused by an external cause. Further definitions refer to experiencing harm, injury, or loss (4). These definitions indicate that an accident at work is a sudden, unplanned, single event that causes injury or ill health (leads to physical harm), is work-related (5–13). An overview of selected definitions of occupational accidents used in different countries is summarized collectively in Table 1. For the purpose of the analyses, a definition was adopted that takes into account the four characteristics of an event that must occur simultaneously for an event to be classified as an occupational accident. Thus, an accident at work will be a sudden event, caused by an external cause, resulting in injury or death of a worker and related to the work performed (14). The characteristics of an accident at work that occur require interpretation, whereby:

- A sudden event is defined as an event that does not last longer than one work shift.
- An external cause—an event is considered an accident if it occurred as a result of external factors.
- An injury is damage to body tissue or human organs.
- Work-related—while an employee is performing activities for the employer, including activities without the employer’s instructions.

**Table 1:** Overview of work accident definitions.

Authors (year)/country	Defining an accident at work
Manu et al. (2012)/United Kingdom (5)	An accident is any unplanned event that causes injury or ill health to people, damage to or loss of plant property, materials or the environment, or loss of business opportunities.
Castaldo et al. (2024)/Italy (9)	A work accident is defined as a single occurrence in the course of work that leads to physical or mental harm.
Herrera-Pérez et al. (2023)/Spain (8)	A work accident is any injury sustained by an employee as a result of or in connection with work performed for hire.
Dyreborg et al. (2022)/Denmark (7)	An accident at work is a separate, sudden, and unexpected event in the course of work that leads to physical harm (injury). The phrase “in the course of work” is understood to mean an event during the performance of work activities or during the time spent at work and includes traffic accidents occurring in the course of work.
Zakaria et al. (2012)/Malaysia (6)	A work accident is an unplanned and uncontrolled event in which the action or reaction of an object, substance, person, or radiation causes injury or the likelihood of injury.
Nowacki (2021)/EU (10)	An accident at work is any sudden occurrence at work that causes physical or psychological injury (European labor market).
Ivascu and Cioca (2019)/EU (11)	An accident at work is a sudden event that occurs during organizational activities and results in physical or psychological injury to a worker (European labor accident statistics).
Paguay et al. (2023)/Ecuador (12)	An accident at work is an unexpected event resulting in physical or mental injury, illness, or death.
Garus-Pakowska et al. (2017)/Poland Act, 2002 (13)	An accident at work is an event that meets four elements: the suddenness of the event, the externality of the cause, the relationship of the event to work, and the result in the form of damage such as injury or death.

The issue of accidents at work plays a significant role among researchers in various countries. Ongoing studies in the field of accident rates focus on (Table 2), among other things:

- Studying accidents occurring in various industries (1, 15–17).
- Determining and analyzing accident measures, applying statistical methods to accident studies (10, 12, 18–20).
- Taking into account the timeframe of accident events (day of the week, time of occurrence of an occupational accident), time of occupational absenteeism (15, 21–25).
- Consideration of demographic characteristics of accident victims (15).
- Analysis of the causes of the accident (24).
- Analyses of the location of the injury (21, 23).
- Implementation of measures to reduce the occurrence of accidents (16, 20).
- Application of methods to support safety management in reducing accidents at work (26, 27).
- Application of AI methods (28).

**Table 2:** A review of research in the field of occupational accident issues.

Authors (year)	Research directions in the field of occupational accident issues
Ceylan (2012) (1)	Identifying the groups most frequently involved in accidents, their locations, and their causes. Analysis of accident rates.
Fontaneda et al. (2022) (15)	Determining the age groups most frequently involved in accidents at work, determining the time of the incident, the day of the week, the location of the incident and the consequences.
Xu and Xu (2020) (16)	Analysis of the accident rate, taking into account the severity of the consequences. Identification of causes and consequences of incidents. Selection of measures aimed at reducing risks.
Ali et al. (2010) (17)	Descriptive and ranking analysis of the causes of accidents in the construction industry, as well as the proposal of solutions to prevent the occurrence of accidents.
Ghamari et al. (2012) (18)	Study of the relationship between the occurrence of accidents and age, seniority, level of education, shift work. The use of the chi-square test.
Krause (2015) (19)	Analysis of measures of accident rates in the mining industry.
Paguay et al. (2023) (12)	Analysis of the effect of the economic sector on the rate of permanent disability resulting from occupational accidents. Use of the Tukey, Duncan test.
Nowacki (2021) (10)	Assessment of the relative risk RR of fatal accidents in the manufacturing sector in EU countries, and identification of differences in the level of occupational safety depending on GDP.
Małysa (2023) (20)	Analysis of relative risk (RR) in the construction industry. Forecasting the value of relative risk. Proposals for preventive measures in the scope of conducted analysis.
Silva and Jacinto (2012) (21)	Systematic data mining accident exploration. Analyses using statistical apparatus aimed at studying correlations between variables.
Shao et al. (2019) (22)	Studying patterns of fatal accidents using correlation coefficient analysis and analysis of variance. Identification of month, time, cause of fatal accidents.
Nag and Patel (1998) (23)	Determining the severity of the incidents, the location of the injury, the time period for recording the most frequent accident incidents (June-July), the shift on which the accident occurred.
Hintikka and Saarela (2010) (25)	Analysis of violent occupational accidents determined by analyzing statistical data on the description of the accident event.
Machado et al. (2023) (26)	Analysis of occupational hazards and the role of risk assessment and preventive measures in improving occupational safety. Selection of methods, tools to support OSH activities.
Fraqaan Nai’em et al. (2021) (24)	Accident analysis, taking into account the time of accident registration and the demographic characteristics of those injured in accident at work.
Shehadeh and Alshboul (2025) (27)	Predictive analytics using advanced ensemble machine learning algorithms—predict occupational injuries.

Analyses conducted on the issue of accidents at work allow for a better understanding of their causes. This study presents the possibility of applying Pearson correlation analysis, which occurs between the seniority group experiencing accidents at work and the activities and causes that are their source. Analyses conducted in this manner can enable the establishment of priorities for safety-related actions tailored individually to the seniority groups and complement the current state of knowledge in this area. Therefore, the aim of the study is to identify the characteristics of accident events for which the highest values of the Pearson linear correlation coefficient (impact strength) are recorded, in order to determine which activities and causes of accidents dominate in each seniority group.

Therefore, for the purposes of this study, the hypothesis was formulated that it is possible to individually determine the direction of safety-related actions for the studied seniority groups, taking into account the characteristics of the accident events (cause of the accident, activity performed at the time of the accident). This research hypothesis is justified by the nature of the issue of accidents in the steel sector, classified as a heavy industry, due to the specific working conditions and hazards involved. That is why it is so important to identify the characteristics describing accidents that are recorded most often in a given seniority group in order to individualize the direction of pro-safety actions.

## Materials and methods

**Figure 1:**
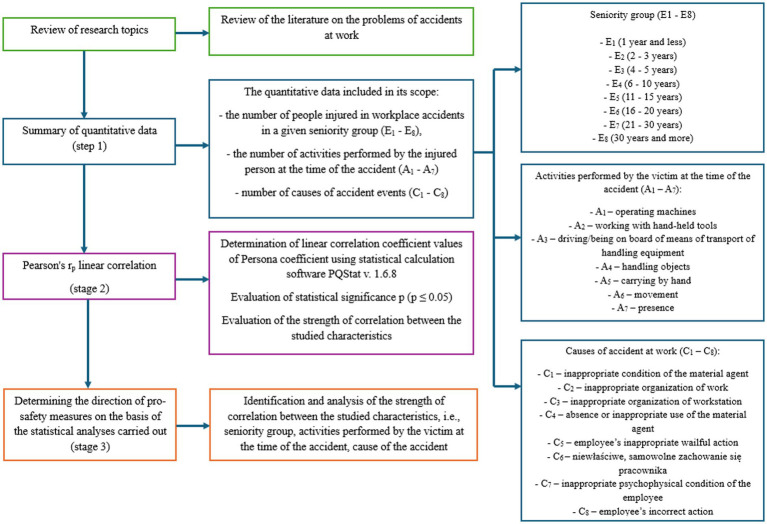
Own research methodology.

The achievement of the study’s stated goal was possible thanks to the development of a four-stage methodology and a review of existing research on accidents at work. In the first stage, quantitative data were compiled on the number of people injured in accident at work in the steel sector in Poland, broken down by seniority. The steel sector was selected based on the specific working conditions and the number of recorded occupational accidents. For the purposes of the analyses, seven seniority groups (E_1_–E_8_) were identified—see Figure 1. Quantitative data were then compiled on the activities performed by the injured person at the time of the accident (A_1_–A_7_) and the causes of accident events (C_1_–C_8_). The statistical data covered a 14-year period (2009–2022). The timeframe is justified by the availability of data published by Statistic Poland (29).

In the second stage of the analyses, Pearson’s linear correlation coefficient values were determined using PQStat v.1.6.8 statistical computing software. Pearson’s linear correlation coefficient values fall within the <−1:1> range. For the purposes of this study, the following interpretation of the coefficient was adopted (30):

- *r_p_* = 1, total positive linear correlation, i.e., an increase in the independent variable is accompanied by an increase in the dependent variable.
- *r_p_* = −1, total negative linear correlation, i.e., an increase in the independent variable is accompanied by a decrease in the dependent variable.
- *r_p_* = 0, indicates no linear correlation between the characteristics studied.

A *t*-test was also conducted to test the significance of the Pearson linear correlation coefficient, which assumed the verification of the following hypotheses (31):

- The null hypothesis (H_0_)—that the Pearson linear correlation coefficient for the studied traits in the population is equal to zero (no linear correlation).
- The alternative hypothesis (H_1_)—that the Pearson linear correlation coefficient for the studied traits in the population satisfies the relationship *r_p_* ≠ 0—a linear correlation exists.

Statistical significance was assessed by comparing the *p*-value to the significance level of *α* = 0.05, adopted for the purposes of this study. Therefore, when:

- *p* ≤ *α*—the null hypothesis (H_0_) of no linear relationship between the variables is rejected in favor of the alternative hypothesis (H_1_)—the result is statistically significant,
- *p* > *α*—there is no basis to reject the null hypothesis (H_0_)—the result is not statistically significant.

In the second stage, the strength of the relationship between the characteristics studied was also determined based on the determined values of the Pearson linear correlation coefficient (*r_p_*). For the purposes of the analyses and determining the correlation strength, the following assumptions were made: *r_p_* ≥ 0.9—very strong correlation (marked +++), 0.7 ≤ *r_p_* ≤ 0.9—strong correlation (marked ++), 0.4 ≤ *r_p_* ≤ 0.7—moderate correlation (marked +).

The third stage of the analyses involved analyzing the results in terms of the strength of correlation between the variables studied and determining the direction of pro-safety actions in the seniority groups being analyzed—individualization of actions.

It should be emphasized that the analysis of correlation between variables involves the statistical assessment of the covariation of two variables—it indicates that changes in one variable are related in a specific way (e.g., linearly) to changes in the other. Correlation is most often measured using Pearson or Spearman correlation coefficients. However, the existence of a correlation does not imply a causal relationship between variables. Causal inference requires the fulfillment of additional criteria, such as: (1) covariation, (2) temporal sequence (the cause precedes the effect), (3) exclusion of alternative cases. Correlation is therefore a necessary, but not sufficient, condition for establishing causality.

## Results

The analyses conducted in the Results section were divided into three areas according to the assumptions presented in the in-house methodology (Figure 1). In the first part of the analyses, Pearson’s linear correlation coefficient values were determined between the seniority groups (E_1_–E_8_) and the activities performed by the injured person at the time of the accident (A_1_–A_8_). Analyses carried out in this way made it possible to obtain an answer to the research question posed: which activity performed by the injured person at the time of the accident shows the strongest correlation with the studied seniority group (RQ-1).

Similarly, the research question posed was also applied to the causes of occurring accident events. Accordingly, the research question was formulated: in terms of which causes of accident events are registered the strongest connection in relation to the studied seniority groups (RQ-2). An important element in the scope of the conducted analyses was also the determination of the value of the linear correlation coefficient *r_p_* between the activity performed by the injured person and the cause of the accident event, allowing to assess the strength of the link between the variables.

The analyses carried out allow the realization of the stated purpose of the work, as well as verification of the established research hypothesis on the possibility of selecting protective prophylaxis for the studied seniority groups, taking into account the individual characteristics of accident events (RH).

### Correlation analysis between the seniority group and the activity performed at the time of the accident

Pearson’s linear correlation analysis was carried out between the seniority groups (E_1_–E_8_) and the activity performed by the injured person at the time of the accident. Accordingly, the values of the linear correlation coefficient *r_p_* were determined using PQStat v.1.6.8 software. The determined values of the correlation coefficient *r_p_* are summarized in Table 3.

**Table 3:** Values of linear correlation coefficient *r_p_*—seniority group/activity.

Seniority group	Physical activity performed by the victim at the time of the accident						
	A_1_	A_2_	A_3_	A_4_	A_5_	A_6_	A_7_
E_1_ (1 year and less)	0.652	0.858	−0.016	0.636	0.745	0.581	0.279
E_2_ (2-3 years)	0.412	0.726	0.175	0.669	0.713	0.644	0.375
E_3_ (4-5 years)	0.469	0.046	−0.073	0.299	0.233	0.323	0.356
E_4_ (6–10 years)	0.578	0.459	0.263	0.508	0.528	0.455	0.455
E_5_ (11–15 years)	0.548	0.315	0.196	0.817	0.567	0.742	0.729
E_6_ (16–20 years)	0.532	0.275	0.316	0.745	0.555	0.796	0.829
E_7_ (21–30 years)	0.536	0.514	0.291	0.860	0.705	0.859	0.804
E_8_ (31 years and more)	0.876	0.496	0.053	0.866	0.727	0.762	0.856

For the determined values of the linear correlation coefficient, an assessment of statistical significance was made by comparing the values of the determined *p* (Table 4), with the values of the level of significance adopted for the purpose of the analyses, *α* = 0.05. According to the assumptions described in the Materials and Method chapter, it was assumed that if *p* ≤ *α* then the result is statistically significant and the null hypothesis H_0_ is rejected in favor of the alternative hypothesis H_1_—the result is statistically significant.

**Table 4:** The *p*-value of the Pearson correlation coefficients significance test.

Seniority group	The *p*-value of the Pearson correlation coefficients						
	A_1_	A_2_	A_3_	A_4_	A_5_	A_6_	A_7_
E_1_ (1 year and less)	**0.012**	**0.000**	0.958	**0.014**	**0.003**	**0.029**	0.334
E_2_ (2-3 years)	0.143	**0.003**	0.550	**0.009**	**0.004**	**0.013**	0.186
E_3_ (4-5 years)	0.090	0.876	0.805	0.298	0.422	0.260	0.212
E_4_ (6–10 years)	**0.030**	0.098	0.363	0.063	0.052	0.102	0.102
E_5_ (11–15 years)	**0.042**	0.273	0.500	**0.001**	**0.034**	**0.002**	**0.003**
E_6_ (16–20 years)	0.050	0.342	0.272	**0.002**	**0.039**	**0.001**	**0.001**
E_7_ (21–30 years)	**0.048**	0.060	0.313	**0.000**	**0.005**	**0.000**	**0.000**
E_8_ (31 years and more)	**0.000**	0.071	0.857	**0.000**	**0.003**	**0.002**	**0.000**

*P* values < 0.05 are in bold.

Based on the analyses, it was found that statistically significant Pearson linear correlations (*p* ≤ 0.05), are positive correlations. Thus, an increase in one variable is accompanied by an increase in the other. Statistically significant correlations were registered between (Table 4):

- E_1_ seniority group (1 year and less) and causes of accidents: A_1_ (operating machines)—*r_p_* = 0.652, *p* = 0.012, A_2_ (working with hand-held tools)—*r_p_* = 0.858, *p* = 0.000, A_4_ (handling of objects)—*r_p_* = 0.636, *p* = 0.014, A_5_ (carrying by hand)—*r_p_* = 0.745, *p* = 0.003, A6 (movement)—*r_p_* = 0.581, *p* = 0.029.
- E_2_ seniority group (2-3 years) registers a positive correlation and is statistically significant for activities: A_2_ (working with hand-held tools)—*r_p_* = 0.726, *p* = 0.003, A_4_ (*r_p_* = 0.669, *p* = 0.009)—handling objects, A_5_ (*r_p_* = 0.713, *p* = 0.004)—carrying by hand, A_6_ (*r_p_* = 0.644, *p* = 0.013)—movement.
- Seniority group E_4_ (6–10 years) registered a positive correlation and statistically significant for activities: A_1_ (*r_p_* = 0.578, *p* = 0.030)—operating machines.
- E_5_ seniority group (11–15 years) registered a positive correlation and statistically significant for activities: A_1_ (*r_p_* = 0.548, *p* = 0.042)—operating machines, A_4_ (*r_p_* = 0.817, *p* = 0.014)—handling of objects, A_5_ (*r_p_* = 0.567, *p* = 0.034)—carrying by hand, A_6_ movement—(*r_p_* = 0.742, *p* = 0.002) and A_7_ (*r_p_* = 0.729, *p* = 0.003)—presence.
- E_6_ (16–20 years) registered a positive correlation and statistically significant for activities: A_4_ (*r_p_* = 0.745, *p* = 0.002)—handling of objects, A_5_ (*r_p_* = 0.555, *p* = 0.039)—carrying by hand, A_6_ movement—(*r_p_* = 0.796, *p* = 0.001) and A_7_ (*r_p_* = 0.829, *p* = 0.001)—presence.
- E_7_ (21–30 years) registered a positive correlation and statistically significant for activities: A_1_ (*r_p_* = 0.539, *p* = 0.048)—operating machines, A_4_ (*r_p_* = 0.860, *p* = 0.000)—handling of objects, A_5_ (*r_p_* = 0.705, *p* = 0.005)—carrying by hand, A_6_ movement—(*r_p_* = 0.859, *p* = 0.000) and A_7_ (*r_p_* = 0.804, *p* = 0.000)—presence.
- E_8_ (31 years and more) registered a positive correlation and statistically significant for activities: A_1_ (*r_p_* = 0.876, *p* = 0.000)—operating machines, A_4_ (*r_p_* = 0.866, *p* = 0.000)—handling of objects, A_5_ (*r_p_* = 0.727, *p* = 0.003)—carrying by hand, A_6_ movement—(*r_p_* = 0.762, *p* = 0.002).

No statistical significance was registered for the E_3_ seniority group (4-5 years), so the data do not provide evidence of a linear and statistically significant relationship *p* > 0.05.

For the determined values of Pearson’s linear correlation coefficient (Table 3), where *p*-values <0.05 were assigned to the strength of the interaction between the variables, according to the assumptions described in Materials and Method. The highest value of linear correlation coefficient *r_p_* was obtained for (Table 5):

- Seniority group E_1_ (1 year and less) and the activity performed by the injured party related to working with hand-held tools A_2_ (*r_p_* = 0.858, *p* = 0.000)—strong correlation (++).
- Seniority group E_2_ (2-3 years) and the activity performed by the injured person related to carrying by hand A_5_ (*r_p_* = 0.713, *p* = 0.004)—strong correlation (++).
- Seniority group E_4_ (6–10 years) and the victim’s activity related to operating machinery A_1_ (*r_p_* = 0.578, *p* = 0.030)—moderate correlation (+).
- Seniority group E_5_ (11–15 years) and the victim’s activity related to handling objects A_4_ (*r_p_* = 0.817, *p* = 0.002)—strong correlation (++).
- Seniority group E_6_ (16–20 years) and the activity performed by the injured person related to attendance (the employee’s performance of normal work activities)—A_7_ (*r_p_* = 0.829, *p* = 0.001)—strong correlation (++).
- Seniority group E_7_ (21–30 years) and the injured worker’s activity related to handling objects A_4_ (*r_p_* = 0.860, *p* = 0.000)—strong correlation (++).
- Seniority group E_8_ (31 years and more) and the activity performed by the injured person related to operating machines A_1_ (*r_p_* = 0.876, *p* = 0.000)—strong correlation (++).

**Table 5:** Strength of association between variables—seniority group/activity.

Seniority group	The strength of the relationship between the variables						
	A_1_	A_2_	A_3_	A_4_	A_5_	A_6_	A_7_
E_1_ (1 year and less)	+	**++**		+	++	+	
E_2_ (2-3 years)		++		+	**++**	+	
E_3_ (4-5 years)							
E_4_ (6–10 years)	**+**						
E_5_ (11–15 years)	+			**++**	+	++	++
E_6_ (16–20 years)				++	+	++	**++**
E_7_ (21–30 years)	+			**++**	++	++	++
E_8_ (31 years and more)	**++**			++	++	++	++

*P* values < 0.05 are in bold.

### Correlation analysis between seniority groups and causes of occupational accidents

Pearson’s linear correlation analysis was also carried out between seniority groups (E_1_–E_8_) and causes of occupational accidents (C_1_–C_8_) described in Figure 1. The statistical software PQStat v.1.6.8 was used for the analyses carried out. The determined values of the correlation coefficient between seniority groups and causes of occupational accidents are summarized collectively in Table 6. The determined values of the correlation coefficient *r_p_* are greater than 0—positive correlation. However, in the case of the correlation between seniority group E_4_ (6–10 years) and C_7_ (improper psychophysical condition of the worker), a negative correlation *r_p_* = −0.162 (*r_p_* < 0) was registered.

**Table 6:** Values of linear correlation coefficient *r_p_*—seniority group/cause of accidents.

Seniority group	Cause of accidents at work							
	C_1_	C_2_	C_3_	C_4_	C_5_	C_6_	C_7_	C_8_
E_1_ (1 year and less)	0.663	0.434	0.663	0.786	0.461	0.855	0.120	0.850
E_2_ (2-3 years)	0.363	0.637	0.339	0.407	0.590	0.574	0.632	0.744
E_3_ (4-5 years)	0.359	0.190	0.532	0.414	0.460	0.299	0.170	0.179
E_4_ (6–10 years)	0.560	0.510	0.501	0.166	0.165	0.367	−0.162	0.423
E_5_ (11–15 years)	0.483	0.671	0.638	0.565	0.686	0.445	0.353	0.551
E_6_ (16–20 years)	0.381	0.574	0.658	0.238	0.418	0.245	0.141	0.407
E_7_ (21–30 years)	0.412	0.729	0.634	0.372	0.574	0.412	0.355	0.611
E_8_ (31 years and more)	0.786	0.782	0.770	0.521	0.584	0.533	0.163	0.649

For the determined values of Pearson’s linear correlation coefficient (Table 6), an assessment of statistical significance was made by comparing the values of the determined *p*-statistic to the significance level *α* = 0.05, adopted for the purpose of the study. According to the assumptions made, it was considered that when *p* ≤ *α*, the result is statistically significant. Thus, the null hypothesis H_0_ is rejected in favor of the alternative hypothesis H_1_—a linear relationship exists, and the result is statistically significant. Table 7 summarizes the values of the *p*-statistic collectively and compares it with the significance level *α*.

**Table 7:** The *p*-value of the Pearson correlation coefficients significance test.

Seniority group	The *p*-value of the Pearson correlation coefficients							
	C_1_	C_2_	C_3_	C_4_	C_5_	C_6_	C_7_	C_8_
E_1_ (1 year and less)	**0.009**	0.121	**0.009**	**0.001**	0.097	**0.000**	0.683	**0.000**
E_2_ (2-3 years)	0.202	**0.014**	0.236	0.149	**0.026**	**0.032**	**0.015**	**0.002**
E_3_ (4-5 years)	0.208	0.267	0.051	0.141	0.098	0.299	0.562	0.541
E_4_ (6–10 years)	**0.037**	0.063	0.068	0.571	0.573	0.197	0.580	0.132
E_5_ (11–15 years)	0.080	**0.009**	**0.014**	**0.037**	**0.007**	0.111	0.216	**0.041**
E_6_ (16–20 years)	0.179	**0.032**	**0.011**	0.413	0.136	0.398	0.631	0.148
E_7_ (21–30 years)	0.143	**0.003**	**0.015**	0.191	**0.032**	0.143	0.213	**0.020**
E_8_ (31 years and more)	**0.000**	**0.001**	**0.001**	0.056	**0.028**	**0.049**	0.578	**0.012**

*P* values < 0.05 are in bold.

The correlations of interest for the analyses conducted were for the following seniority groups and causes of occupational accidents (*p* ≤ 0.05)—positive and statistically significant correlations:

- E_1_ seniority group (1 year and less) and causes: C_1_ (inappropriate condition of the material agent)—*r_p_* = 0.663, *p* = 0.009, C_3_ (inappropriate organization of workstation)—*r_p_* = 0.663, *p* = 0.009, C_4_ (absence or inappropriate use of the material agent)—*r_p_* = 0.786, *p* = 0.001, C_6_ (employee’s inappropriate wailful action)—*r_p_* = 0.855, *p* = 0.003, and C_8_ (employee’s incorrect action)—*r_p_* = 0.850, *p* = 0.000.
- E_2_ seniority group (2-3 years) and causes of occupational accidents: C_2_ (inappropriate organization of work)—*r_p_* = 0.637, *p* = 0.014, C_5_ (*r_p_* = 0.590, *p* = 0.026)—not using protective equipment, C_6_ (employee’s inappropriate wailful action)—*r_p_* = 0. 574, *p* = 0.032, C_7_ (inappropriate psychophysical condition of the employee)—*r_p_* = 0.632, *p* = 0.015, and C_8_ (employee’s incorrect action)—*r_p_* = 0.744, *p* = 0.002.
- Seniority group E_3_ (4-5 years) did not register a significant statistical correlation *p* > 0.05, indicating that there is no association between seniority group and causes of occupational accidents C_1_–C_8_—causes are randomly distributed.
- Seniority group E_4_ (6–10 years) and cause C_1_ (*r_p_* = 0.560, *p* = 0.037)—inappropriate condition of the material agent.
- E_5_ seniority group (11–15 years) and causes of accidents: C_2_ (*r_p_* = 0.671, *p* = 0.009)—inappropriate organization of work, C_3_ (*r_p_* = 0.638, *p* = 0.014)—inappropriate organization of workstation, C_4_ (*r_p_* = 0.565, *p* = 0. 037)—absence or inappropriate use of the material agent, C_5_ (*r_p_* = 0.686, *p* = 0.007)—not using protective equipment, and C_8_ (*r_p_* = 0.551, *p* = 0.041)—employee’s incorrect action.
- Seniority group E_6_ (16–20 years) with accident causes: C_2_ (inappropriate organization of work)—*r_p_* = 0.574, *p* = 0.032 and C_3_ (inappropriate organization of workstation)—*r_p_* = 0.658, *p* = 0.011.
- Seniority group E_7_ (21–30 years) with causes of accident events: C_2_ (inappropriate organization of work)—*r_p_* = 0.729, *p* = 0.003, C_3_ (inappropriate organization of workstation)—*r_p_* = 0.634, *p* = 0.015, C_5_ (*r_p_* = 0.574, *p* = 0.032)—not using protective equipment, and C_8_ (employee’s incorrect action)—*r_p_* = 0.611, *p* = 0.020.
- Seniority group E_8_ (31 years and over) registers correlations with the causes: C_1_ (*r_p_* = 0.786, *p* = 0.000)—improper condition of material factor, C_2_ (*r_p_* = 0.782, *p* = 0.001)—inappropriate organization of work, C_3_ (*r_p_* = 0.770, *p* = 0.001)—inappropriate organization of workstation, C_5_ (*r_p_* = 0.584, *p* = 0.028)—not using protective equipment, C_6_ (*r_p_* = 0.533, *p* = 0.049)—employee’s inappropriate wailful action, and C_8_ (*r_p_* = 0.649, *p* = 0.012)—employee’s incorrect action.

For the tabulated values of Pearson’s linear correlation coefficient, the strength of the relationship between the variables was determined using a three-level scale (very strong, strong, moderate strength). For the seniority group (Table 8):

- E_1_ (1 year and less) there is a strong association (++) with employee’s inappropriate wailful action C_6_—r_p_ = 0.855, *p* = 0.000.
- E_2_ (2-3 years) there is a strong association (++) with an activity relating to employee misbehavior C_8_—*r_p_* = 0.744, *p* = 0.002.
- E_4_ (6–10 years) moderate association (+) with the activity relating to the improper condition of the material factor—C_1_ (*r_p_* = 0.560, *p* = 0.037).
- E_5_ (11–15 years) registers a moderate association (+) with activity C_5_ (*r_p_* = 0.686, *p* = 0.007)—employee’s failure to use protective equipment.
- E_6_ (16–20 years) registers a moderate association with activity C_2_ (*r_p_* = 0.574, *p* = 0.032)—improper work organization.
- E_7_ (21–30 years) registers a strong association with activity C_2_ (*r_p_* = 0.729, *p* = 0.003)—improper work organization.
- E_8_ (31 years and older) registers a strong association with activity C_1_ (*r_p_* = 0.786, *p* = 0.000)—improper condition of material factor.

**Table 8:** Strength of relationship between variables—seniority group/cause of accidents.

Seniority group	The strength of the relationship between the variables							
	C_1_	C_2_	C_3_	C_4_	C_5_	C_6_	C_7_	C_8_
E_1_ (1 year and less)	+		+	++		**++**		++
E_2_ (2-3 years)		+			+	+	+	**++**
E_3_ (4-5 years)								
E_4_ (6–10 years)	**+**							
E_5_ (11–15 years)		+	+	+	**+**			+
E_6_ (16–20 years)		+	**+**					
E_7_ (21–30 years)		**++**	+		+			+
E_8_ (31 years and more)	**++**	++	++		+	+		+

*P* values < 0.05 are in bold.

### Correlation analysis between activity and causes of work accidents

Pearson’s linear correlation analyses were also conducted between causes of accident events (C_1_–C_8_) and activities performed by the injured person at the time of the accident (A_1_–A_7_). The analyses conducted were aimed at assessing the presence of a relationship between the variables, as well as determining the strength of the association (value of Pearson’s linear correlation coefficient)—according to the assumptions described in the Materials and Method section. Collectively, the values of Pearson’s correlation coefficient between the studied variables are summarized in Table 9. The determined values indicate a positive correlation *r_p_* > 0. A negative correlation was registered between the activity of driving or riding means of transport or operating moving machinery (A_3_) and the cause of the accident C_1_—operation of machines. In the following section, the statistical significance of *p* ≤ *α* was evaluated.

**Table 9:** Values of linear correlation coefficient r_p_—activity/cause of accidents.

Activity performed by the injured party	Causes of accidents at work							
	C_1_	C_2_	C_3_	C_4_	C_5_	C_6_	C_7_	C_8_
A_1_	0.860	0.749	0.750	0.688	0.592	0.737	0.078	0.788
A_2_	0.580	0.691	0.647	0.689	0.636	0.841	0.362	0.913
A_3_	−0.037	0.276	0.227	0.026	0.305	0.228	0.167	0.159
A_4_	0.759	0.755	0.841	0.705	0.658	0.705	0.293	0.823
A_5_	0.604	0.801	0.757	0.642	0.701	0.731	0.416	0.851
A_6_	0.589	0.721	0.756	0.531	0.671	0.679	0.455	0.789
A_7_	0.543	0.561	0.578	0.357	0.364	0.313	0.102	0.488

Next, the statistical significance between the studied variables was assessed by comparing the *p*-values to the significance level of *α* = 0.05 adopted for the analysis. For these analyses, the null hypothesis H_0_ was rejected in favor of the alternative hypothesis H_1_ in the case of a linear relationship between the variables—the result was statistically significant when *p* < *α*—Table 10.

**Table 10:** *P*-values of the Pearson correlation coefficient significance test.

Activity performed by the injured party	The *p*-value of the Pearson correlation coefficients							
	C_1_	C_2_	C_3_	C_4_	C_5_	C_6_	C_7_	C_8_
A_1_	**0.000**	**0.002**	**0.002**	**0.007**	**0.026**	**0.026**	0.079	**0.001**
A_2_	**0.029**	**0.006**	**0.012**	**0.006**	**0.015**	**0.000**	0.203	**0.000**
A_3_	0.898	0.339	0.435	0.927	0.289	0.433	0.569	0.587
A_4_	**0.002**	**0.002**	**0.000**	**0.005**	**0.011**	**0.005**	0.309	**0.000**
A_5_	**0.022**	**0.001**	**0.002**	**0.013**	**0.005**	**0.003**	0.139	**0.000**
A_6_	**0.026**	**0.004**	**0.002**	0.051	**0.008**	**0.008**	0.102	**0.001**
A_7_	0.045	**0.037**	**0.030**	0.211	0.201	0.275	0.729	0.077

*P* values < 0.05 are in bold.

The correlations analyzed in relation to determining the direction of actions to improve occupational safety referred to:

- Activities related to the operation of machines A_1_, during which the causes of accidents were recorded related to: inappropriate condition of the material agent (*r_p_* = 0.860, *p* = 0.000)—C_1_, inappropriate organization of work (*r_p_* = 0.749 *p* = 0.002)—C_2_, inappropriate organization of workstation (*r_p_* = 0.750, *p* = 0.002)—C_3_, absence or inappropriate use of the material agent C_4_ (*r_p_* = 0.688, *p* = 0.007), not using protective equipment C_5_—(*r_p_* = 0.592, *p* = 0.026), employee’s inappropriate wailful action C_6_ (*r_p_* = 0.737, *p* = 0.026), employee’s incorrect action C_8_—(*r_p_* = 0.788, *p* = 0.001).
- Activities related to working with hand-held tools A_2_, during which the causes of accidents were recorded as being related to: inappropriate condition of the material agent (*r_p_* = 0.580, *p* = 0.029)—C_1_, inappropriate organization of work (*r_p_* = 0.691, *p* = 0.006)—C_2_, inappropriate organization of workstation (*r_p_* = 0.647, *p* = 0.012)—C_3_, absence or inappropriate use of the material agent C_4_ (*r_p_* = 0.689, *p* = 0.006), not using protective equipment C_5_ (*r_p_* = 0.636, *p* = 0.015), employee’s inappropriate wailful action C_6_ (*r_p_* = 0.841, *p* = 0.000), and employee’s incorrect action C_8_—(*r_p_* = 0.913, *p* = 0.000).
- Activity related to handling of objects A_4_, during which the causes of accidents were recorded related to: inappropriate condition of the material agent (*r_p_* = 0.759, *p* = 0.002)—C_1_, inappropriate organization of work (*r_p_* = 0.755, *p* = 0.002)—C_2_, inappropriate organization of workstation (*r_p_* = 0.841, *p* = 0.000)—C_3_, absence or inappropriate use of the material agent C_4_ (*r_p_* = 0.705, *p* = 0.005), not using protective equipment C_5_ (*r_p_* = 0.658, *p* = 0.011), employee’s inappropriate wailful action C6 (*r_p_* = 0.705, *p* = 0.005), employee’s incorrect action C_8_—(*r_p_* = 0.823, *p* = 0.000).
- Activity related to manual transport A_5_, during in which the causes of accidents were recorded as being related to: inappropriate condition of the material agent (*r_p_* = 0.604, *p* = 0.022)—C_1_, inappropriate organization of work (*r_p_* = 0.801, *p* = 0.001)—C_2_, inappropriate organization of workstation (*r_p_* = 0.757, *p* = 0.002)—C_3_, absence or inappropriate use of the material agent C_4_ (*r_p_* = 0.642, *p* = 0.013), not using protective equipment C_5_ (*r_p_* = 0.701, *p* = 0.005), employee’s inappropriate wailful action C_6_ (*r_p_* = 0.731, *p* = 0.003), employee’s incorrect action C_8_—(*r_p_* = 0.851, *p* = 0.000).
- Activity related to movement A_6_, during which the causes of accidents were recorded as being related to: inappropriate condition of the material agent (*r_p_* = 0.589, *p* = 0.026)—C_1_, inappropriate organization of work (*r_p_* = 0.721, *p* = 0.004)—C2, inappropriate organization of workstation (*r_p_* = 0.756, *p* = 0.002)—C_3_, not using protective equipment C5 (*r_p_* = 0.671, *p* = 0.008), employee’s inappropriate wailful action C_6_ (*r_p_* = 0.679, *p* = 0.008), employee’s incorrect action C_8_—(*r_p_* = 0.789, *p* = 0.001).
- Activity related to presence at work A_7_, during which causes of accidents were recorded related to inappropriate organization of work (*r_p_* = 0.561, *p* = 0.037)—C_2_, inappropriate organization of workstation (*r_p_* = 0.578, *p* = 0.030)—C_3_.

For activity A_3_ related to driving or riding in a means of transport and causes C_1_–C_8_, a statistically insignificant correlation was observed (*p* > 0.05). The data presented do not provide evidence of a linear relationship between the variables.

For the compiled values of the linear correlation coefficient (Table 9), the strength of influence was determined using the assumed three-point scale. The strongest correlations (highest *r_p_* values) were recorded between (Table 11):

- Activity related to operating machinery A_1_—there is a strong correlation (++) with the cause of the accident C_1_—inappropriate condition of the material agent (*r_p_* = 0.860, *p* = 0.000).
- Activity related to working with hand-held tools A_2_—there is a very strong correlation (+++) with the cause related to employee’s incorrect action C_8_—(*r_p_* = 0.913, *p* = 0.000).
- Activity related to handling of objects A_4_—there is a very strong correlation (++) with the cause related to inappropriate organization of workstation C_3_—(*r_p_* = 0.841, *p* = 0.000).
- Activity related to carrying by hand A_5_—there is a strong correlation (++) with the cause related to employee’s incorrect action C_8_—(*r_p_* = 0.851, *p* = 0.000).
- Activity related to movement A_6_ has a strong correlation (++) with the cause related to employee’s incorrect action C_8_ (*r_p_* = 0.789, *p* = 0.001).
- Activity related to presence A_7_ presence has a moderate correlation (+) with the cause related to inappropriate organization of workstation C_3_ (*r_p_* = 0.578, *p* = 0.030).

**Table 11:** Strength of correlation between variables—activity/cause of accidents.

Activity performed by the injured party	The strength of the relationship between the variables							
	C_1_	C_2_	C_3_	C_4_	C_5_	C_6_	C_7_	C_8_
A_1_	**++**	++	++	+	+	++		++
A_2_	+	+	+	+	+	++		**+++**
A_3_								
A_4_	++	++	**++**	++	+	++		++
A_5_	+	++	++	+	++	++		**++**
A_6_	+	++	++		++	++		**++**
A_7_		+	**+**					

*P* values < 0.05 are in bold.

## Analysis and discussion of results

### Correlation analysis between the examined characteristics

The correlation analyses conducted allowed us to determine the linear relationship between the studied variables, as well as the strength of the interaction between them. The study divided employees into seven seniority groups (E_1_–E_7_), considered the causes of accidents (C_1_–C_8_), and the activities performed by the injured person at the time of the accident (A_1_–A_7_).

As part of the correlation analyses conducted between the seniority groups (E_1_–E_7_) and the activities performed by the injured person at the time of the accident (A_1_–A_7_), a relationship was found between the studied variables. These correlations varied in the strength of the interaction between the studied variables (*r_p_* values), but they provided basic information about which seniority groups and which activities performed by the injured person were the source of the accident at work. The determined coefficient values indicated a strong correlation (++), meeting the assumption described in the methodology: 0.7 ≤ *r_p_* ≤ 0.9. Analyzing the repeatability of causes across age groups, it was noted that the most common activities associated with accidents at work included: A_1_—operating machines (5 times), A_4_—handling of objects (6 times), A_5_—carrying by hand (6 times), A_6_—movement (6 times), and A_7_—employee presence (4 times). A key element in improving occupational safety, considering the division of employees into seniority groups, was selecting the highest values of linear correlation coefficients—indicating the strength of the relationship between variables. A selection was made for each seniority group, and based on the analysis, it was determined that each seniority group recorded a different activity performed by the injured person at the time of the accident. The repeatability of activities performed by injured persons in A_4_–A_7_ accidents is particularly noticeable for seniority groups E_5_–E_8_, which differ in the strength of the correlation.

Correlation analysis for seniority groups (E_1_–E_7_) and causes of accidents at work (C_1_–C_8_) suggests that particular attention should be paid to seniority groups E_1_ (1 year or less) and E_8_ (31 years or more), where strong correlations are observed with most of the activities performed by the injured person at the time of the accident. This may be due to lack of experience, awareness of hazards, or lack of compliance with occupational health and safety regulations—group E_1_. In the case of seniority group E_8_, the recording of accidents during specific activities for which a linear, statistically significant relationship was recorded (*p* < 0.05) may result from health status, physical abilities, and familiarity with hazards. When considering the correlation between seniority groups and causes of accidents, individualized preventive measures should be planned, taking into account the employee’s knowledge and mental and physical abilities.

Based on the Pearson linear correlation analysis between the activities performed by the injured person at the time of the accident (A_4_–A_7_) and the causes of the accident (C_1_–C_8_), a strong correlation was observed, and in one case, a very strong one (A_2_–C_8_), which may play a significant role in the implementation of preventive measures. Therefore, it is worth considering building a model that summarizes the strongest correlations in the system: seniority group E—activity performed at the time of the accident A—cause of the accident at work C.

### Correlation analysis in the system of seniority group E-activity A-cause C (E-a-C)

**Figure 2:**
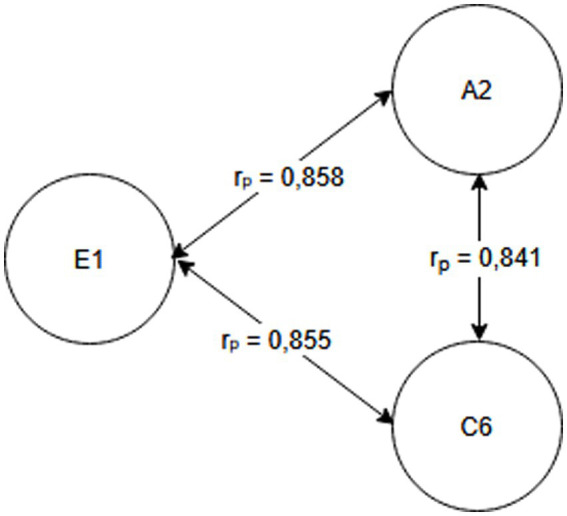
Correlations in the E_1_ seniority group.

**Figure 3:**
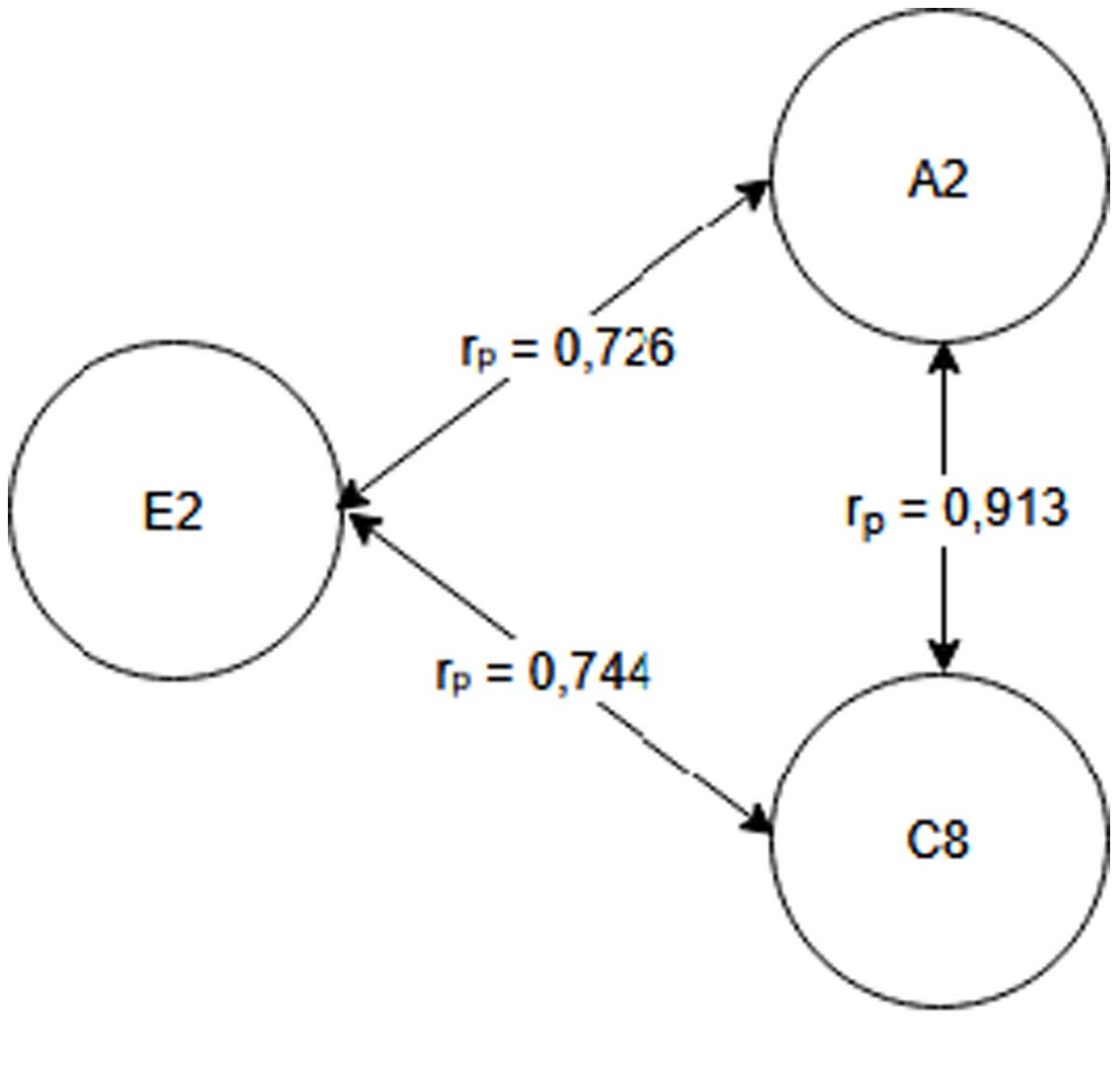
Correlations in the E_2_ seniority group.

**Figure 4:**
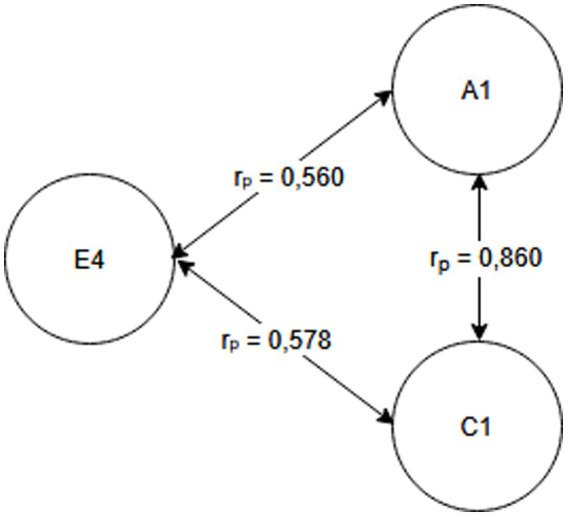
Correlations in the E_4_ seniority group.

**Figure 5:**
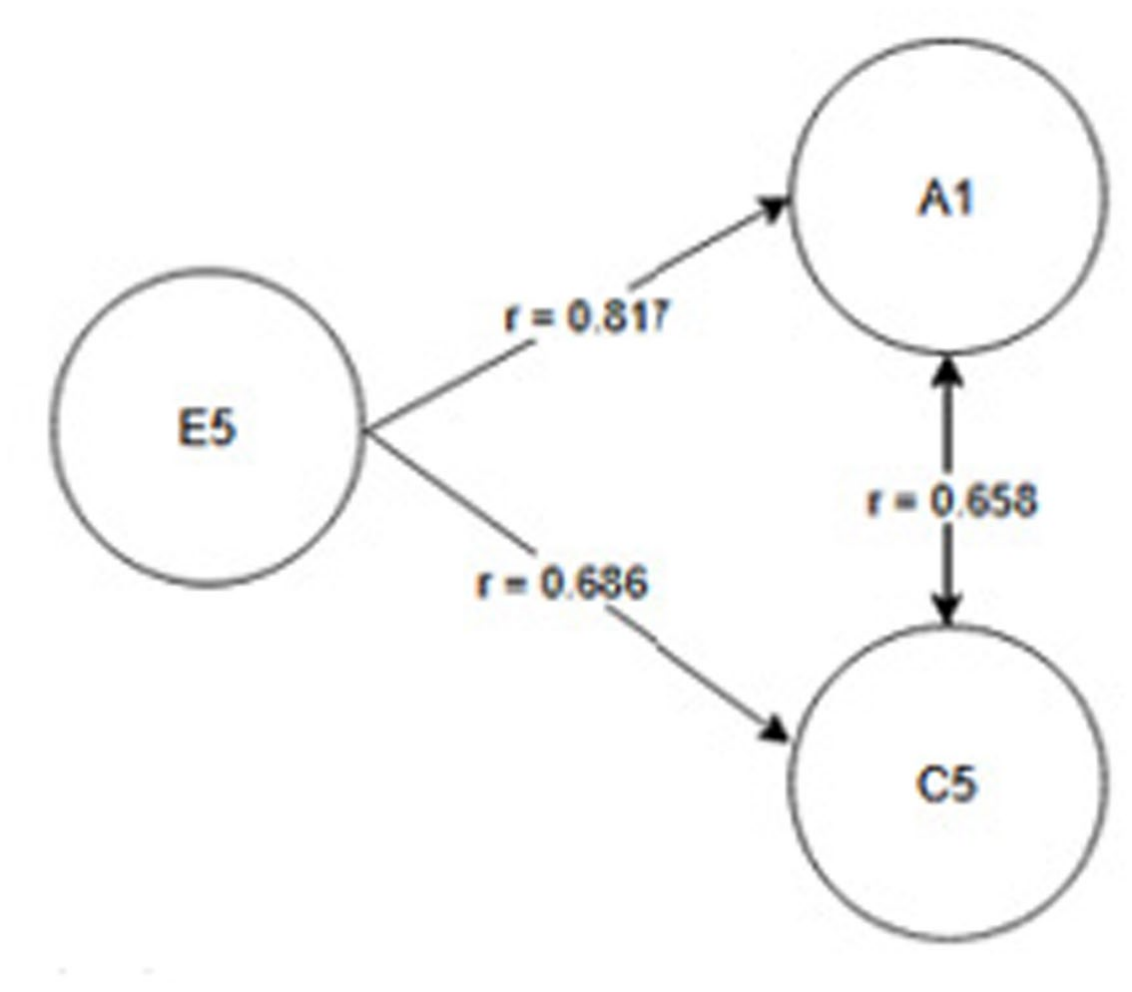
Correlations in the E_5_ seniority group.

**Figure 6:**
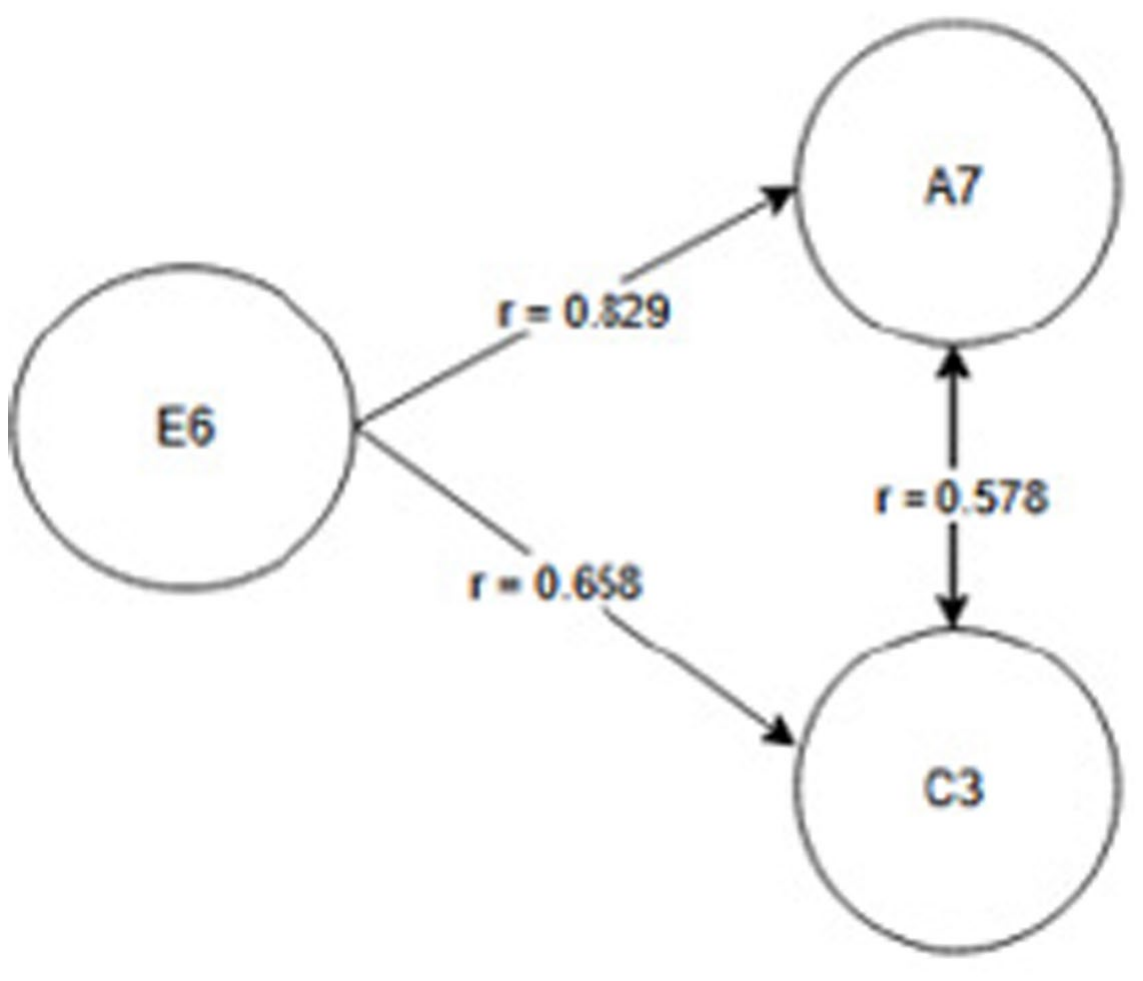
Correlations in the E_6_ seniority group.

**Figure 7:**
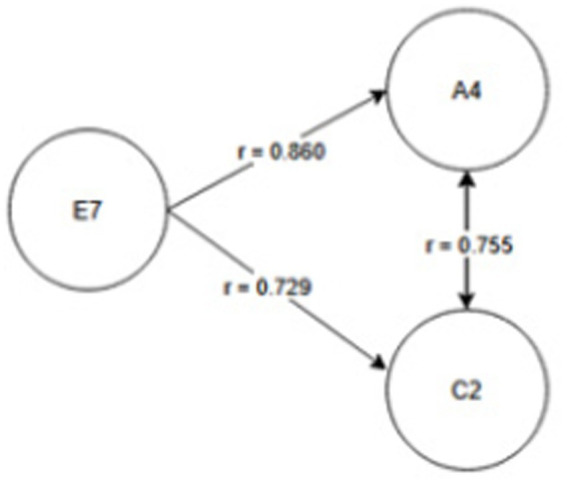
Correlations in the E_7_ seniority group.

**Figure 8:**
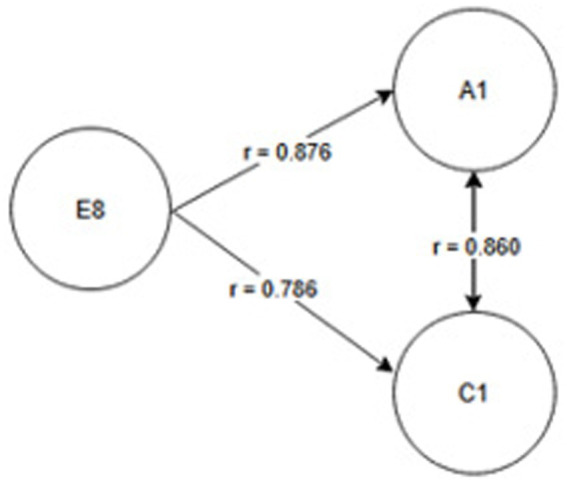
Correlations in the E_8_ seniority group.

A fundamental element in the analysis of occupational accidents is conducting analyses from which conclusions will contribute to eliminating and reducing the number of accidents occurring. This study presents correlation analyses between variables, which allow for the assessment of the strength of the relationship between the examined characteristics describing accident events. For more extensive analyses, the determined correlations were used to build a model combining the seniority group, the activity performed by the injured person at the time of the accident, and the cause of the accident. For each of the seniority groups adopted for the study (E_1_–E_7_), an individual model was built based on the strength of the correlations between the examined characteristics characterizing accident events. Therefore, the strongest correlations were recorded between:

- Seniority group E_1_ (1 year or less) and A_2_ activity related to working with hand tools and the activity performed at the time of the accident, C_6_—inappropriate, arbitrary behavior by the employee. Therefore, safety measures should be implemented regarding specific occupational health and safety regulations regarding working with hand tools, also taking into account the work performed by employees during work—Figure 2.
- Seniority group E_2_ (2-3 years) and activity A_2_ related to working with hand tools and cause C_8_ related to employee misconduct. Therefore, special attention should be paid to the hazards occurring during work with hand tools and supervisors should increase supervision of these activities, as the strongest correlation between these characteristics is related to employee misconduct—Figure 3.
- Seniority group E_4_ (6–10 years), and activity A_1_ related to operating machines, with cause C_1_ being improper condition of the material factor (design defects, overuse). The strongest correlations in this system indicate that accidents in this group occur during the operation of machinery, where the problem lies with the material factor. Therefore, technical, and organizational solutions should be implemented—repairs, maintenance, and employees should be alerted to possible hazards. Supervisor supervision will also be an important element in this regard—Figure 4.
- Seniority group E_5_ (11–15 years), and activity A_1_ related to operating machines and C_5_ related to not using protective equipment (failure to use or use protective equipment correctly, bypassing or disabling fixed and movable guards or other measures limiting and protecting against accident risks). Therefore, in this seniority group, special attention should be paid to work performed on machinery and the employees’ use of measures designed to protect against hazards (technical and organizational protective measures)—Figure 5.
- Seniority group E_6_ (16–20 years), and activity A_7_ related to employee presence and cause C_3_ related to inappropriate organization of workstation. Therefore, in this seniority group, accidents primarily occur due to improper workstation organization, which results from improper placement and storage of work items, failure to remove unnecessary items, etc. Therefore, increased supervision of organizational activities is necessary—Figure 6.
- Seniority group E_7_ (21–30 years) and activity A_4_ related to handling of objects, and cause C_2_—inappropriate organization of work. Improper work organization is related to the performance of work with a small crew, the possibility of admitting an employee with medical contraindications or lack of up-to-date medical examinations, and the lack of occupational health and safety procedures and instructions. Therefore, actions aimed at improving occupational safety should be implemented in this area—Figure 7.
- Seniority group E_8_ (30 years and older) and activity A_1_ related to operating machines, and C_1_ related to inappropriate condition of the material (design defects, improper structure, inadequate strength, excessive wear). Therefore, special attention should be paid to the work carried out on machines and their technical condition, and this also applies to the equipment that enables the implementation of specific production tasks—Figure 8.

By analyzing occupational accidents and taking into account the characteristics of occupational accidents in a given seniority group, it is possible to better understand the recorded events and more accurately select preventive solutions to reduce the risk of accidents.

## Conclusion

Occupational health and safety is an important issue for researchers worldwide, particularly in the area of workplace accidents (1, 23, 24). Analyses focus on better understanding the occurrence of accidents and, above all, providing recommendations for their reduction (20, 25). This article presents the possibility of using Pearson linear correlation analysis to determine the relationship between the studied characteristics (seniority, cause, activity). For the purposes of this study, a research hypothesis was formulated regarding the possibility of selecting preventive measures individually for the studied seniority groups, taking into account the characteristics of accident events. The conducted analyses confirmed the validity of the hypothesis. By taking into account the characteristics of the accident event and the strength of correlations between the characteristics, it was possible to develop a customized model, allowing for determining the direction of pro-safety actions (Figures 2–8), and based on this, the selection of solutions dedicated to the studied seniority group.

The research questions addressed the possibility of determining (identifying) the activity performed by the injured person that demonstrates the strongest correlation with a given seniority group, as well as the causes of accidents. Based on the analyses, the most frequently recorded activities were A_1_ related to operating machines (three times) and A_2_ related to working with hand-held tools (twice). Therefore, these activities should be particularly relevant to the design of safety-oriented activities. However, the most prevalent causes of accidents were: C_8_—employee’s incorrect action (twice) and C_1_—inappropriate condition of the material agent (twice).

Selecting the strongest influences between the studied characteristics allows for prioritizing occupational health and safety (OHS) actions for specific seniority groups (E_1_–E_8_). Therefore, when determining the directions of OHS actions for each seniority group, it is necessary to identify the causes and activities for which the strongest correlations are recorded. Analyses conducted in this way can be an effective tool in implementing actions to reduce the number of recorded accident events in the enterprise.

Accidents at work are recorded every day, making it crucial to better understand their occurrence and seek solutions aimed at reducing them. This is a significant issue because emerging technologies and solutions can also generate new risks for employees (32, 33). Therefore, it is crucial to analyze and better understand the characteristics of accidents at work to effectively identify the causes of recorded incidents and implement effective preventive measures.

The presented analyses demonstrate the potential application of statistical methods to occupational health and safety issues. They do not constitute a risk-reduction solution, but rather a path for preliminary analysis of where to begin and where the problem lies. The effectiveness of implemented prevention should be adequate to the identified occupational hazards and therefore will depend on the underlying causes and consequences of accidents.

## Data Availability

The datasets presented in this study can be found in online repositories. The names of the repository/repositories and accession number(s) can be found at: https://stat.gov.pl/obszary-tematyczne/rynek-pracy/warunki-pracy-wypadki-przy-pracy/wypadki-przy-pracy-w-2024-r-dane-wstepne.3.58.html.
